# Warfarin Dosing in a Patient with *CYP2C9^∗^3^∗^3* and *VKORC1-1639 AA* Genotypes

**DOI:** 10.1155/2014/413743

**Published:** 2014-01-22

**Authors:** Mark Johnson, Craig Richard, Renee Bogdan, Robert Kidd

**Affiliations:** ^1^Bernard J. Dunn School of Pharmacy, Shenandoah University, Winchester, VA 22601, USA; ^2^PinnacleHealth, Harrisburg, PA 17109, USA

## Abstract

Genetic factors most correlated with warfarin dose requirements are variations in the genes encoding the enzymes cytochrome P450 2C9 (CYP2C9) and vitamin K epoxide reductase (VKOR). Patients receiving warfarin who possess one or more genetic variations in *CYP2C9* and *VKORC1* are at increased risk of adverse drug events and require significant dose reductions to achieve a therapeutic international normalized ratio (INR). A 74-year-old white female with atrial fibrillation was initiated on a warfarin dose of 2 mg PO daily, which resulted in multiple elevated INR measurements and three clinically significant hemorrhagic events and four vitamin K antidote treatments over a period of less than two weeks. Genetic analysis later revealed that she had the homozygous variant genotypes of *CYP2C9∗3∗3* and *VKORC1-1639 AA*. Warfarin dosing was subsequently restarted and stabilized at 0.5 mg PO daily with therapeutic INRs. This is the first case report of a white female with these genotypes stabilized on warfarin, and it highlights the value of pharmacogenetic testing prior to the initiation of warfarin therapy to maximize efficacy and minimize the risk of adverse drug events.

## 1. Introduction

Warfarin is the most widely used anticoagulant in the world and has been consistently shown to be effective at preventing emboli in patients with prosthetic heart valves or atrial fibrillation [1]. Achieving a safe and effective warfarin maintenance dose can take weeks or months after the initiation of therapy due to its narrow therapeutic range and wide interindividual dose variation [2]. Unexpected sensitivity to warfarin commonly results in prolonged bleeding caused by excessive anticoagulation and warfarin is the number one cause of hospitalization due to an adverse drug event in the USA [3].

Clinical factors including age, height, weight, gender, race, diet, smoking, comorbidities, prosthetic heart valve, and other medications contribute to the dose variability of warfarin, but genetic factors have been shown to be the largest contributor to the dose variability of warfarin [2, 3]. The two genetic factors that are most correlated with warfarin dose requirements are variations in the genes encoding the enzymes cytochrome P450 2C9 (CYP2C9) and vitamin K epoxide reductase (VKOR) [1].

CYP2C9 is the primary enzyme responsible for inactivating warfarin. The CYP2C9*3 variant allele has been shown to cause an 80% decrease in enzymatic activity of CYP2C9 and therefore contributes to the dose variance of warfarin [4]. The pharmacological target for warfarin is inhibition of the VKOR enyzme, a product of the *VKORC1* gene [5]. The VKOR enzyme reduces vitamin K 2,3-epoxide to the active vitamin K hydroquinone which is a required cofactor in the production of several procoagulation factors [6]. The *VKORC1*-*1639  G* > *A* gene variant results in a 50% decreased transcription of the *VKOR* gene and increases a patient's sensitivity to warfarin [7]. Therefore, the *VKORC1-1639 A* variant contributes significantly to the variability of warfarin dosing [2]. Patients who are homozygous variant for CYP2C9*3 and homozygous variant for *VKORC1-1639 A* are expected to be extremely sensitive to warfarin. Therefore, these patients will require very low doses of warfarin to achieve appropriate therapeutic effect and minimize risk of adverse drug events.

The first detailed clinical report of an individual patient with a genotype of CYP2C9*3*3 and *VKORC1-1639 AA* was a Japanese female stabilized on a maintenance dose of warfarin that was about 90% less than the average starting dose for warfarin [8]. Herein, we provide the first case report of a white female with a genotype of CYP2C9*3*3 and *VKORC1-1639 AA* stabilized on the same maintenance dose of warfarin. This report details the clinical course of the patient, summarizes findings from related case reports, and highlights the value of pharmacogenetic testing for warfarin patients.

## 2. Case Report

### 2.1. Clinical Course

The patient was a 74-year-old (height 157.5 cm and weight 54 kg) white female of Ashkenazi Jewish descent. Her past medical history was significant for atrial fibrillation, hypertension, diabetes mellitus, coronary artery disease, cardiomyopathy, hypothyroidism, myelodysplastic syndrome with chronic anemia, cerebrovascular accident, chronic kidney disease, peptic ulcer disease, peripheral vascular disease, and pulmonary hypertension. The patient reported a previous “hypersensitivity” to warfarin six years earlier at a different institution. Medications included aspirin 81 mg PO daily, isosorbide mononitrate 40 mg PO daily, furosemide 10 mg alternating with 20 mg PO daily, ramipril 10 mg PO daily, amiodarone 200 mg PO daily, atorvastatin 80 mg PO daily, metoprolol 25 mg PO daily, multiple vitamin PO daily, insulin glargine 14 units subcutaneously daily, epoetin alfa 40,000 units subcutaneously weekly, levothyroxine 100 mcg PO daily, calcitriol 0.25 mcg alternating with 0.5 mcg PO daily, and polysaccharide iron complex 150 mg PO twice daily. Due to a recurrence of atrial fibrillation, the patient was initiated on warfarin (Jantoven) at 2 mg PO daily with an international normalized ratio (INR) goal of 2.0–3.0 which would indicate the appropriate level of anticoagulation for this patient.

**Figure 1:**
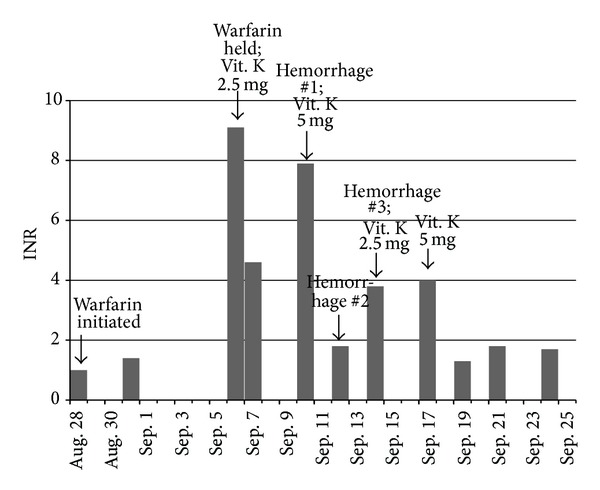
Timeline of INR and significant events after patient was initiated on warfarin 2 mg per day.

The timeline of the subsequent clinical course is outlined in Figure 1. Three days after initiation of warfarin, the patient's INR was 1.4 and the dose remained unchanged. Six days later the patient's INR was 9.1, so warfarin was held and phytonadione (vitamin K) 2.5 mg PO was administered as an antidote to counteract the excessive anticoagulation. One day later the INR had fallen to 4.6, and no additional vitamin K was administered. However, three days later the INR had risen to 7.9, and the patient reported bleeding from her left hand for one hour after accidentally hitting it. As a result, vitamin K was administered a second time at a dose of 5 mg PO. Two days later the INR had decreased to 1.8, yet the patient reported her lip bleeding for approximately 30 minutes after accidentally biting it. Two days later the INR had risen to 3.8, and the patient reported her elbow bleeding for an undetermined period of time after injuring it. Vitamin K was then administered a third time at a dose of 2.5 mg PO. Three days later, the INR had risen to 4.0 and vitamin K was administered a fourth time at a dose of 5 mg PO. Upon subsequent follow-up the INR had decreased to 1.3 after two more days and increased to 1.8 over the next two days, but it then decreased three days later to 1.7. At this time, the decision was made to not restart warfarin due to the supratherapeutic response and to increase the aspirin dose to 325 mg PO daily.

Due to this hypersensitivity to warfarin, the patient consented to genetic testing which revealed CYP2C9*3*3 and *VKORC1-1639 AA* genotypes. The patient received a second opinion, and it was decided to restart warfarin to decrease risks of a thromboembolic event. Seven months after the previous discontinuation of warfarin, a baseline INR of 1.0 confirmed that the patient's untreated INR was not elevated. Warfarin was then reinitiated at 0.5 mg PO two times weekly and slowly titrated up to 0.5 mg PO daily over the next three months to achieve therapeutic INRs. No hemorrhages were reported during this time.

### 2.2. Genetic Analyses

This study was approved by the Shenandoah University Institutional Review Board. The patient signed an informed consent and provided a cheek swab sample for DNA collection at the time of a scheduled appointment. Genomic DNA was isolated from the cheek swab sample using a Qiagen QiaCube automated DNA isolation instrument (Qiagen Inc, Chatsworth, CA) following the manufacturer's protocol and then frozen at −20°C until the time of genotyping. The DNA sample was genotyped for CYP2C9*2 (rs1799853), *3 (rs1057910), *5 (rs28371686), *6 (rs9332131), *7 (rs67807361), *8 (rs7900194), *9 (rs2256871), *11 (rs28371685), *12 (rs9332239), *13 (rs72558187), and *VKORC1-1639 G* > *A* (rs9923231). All genotyping was performed by allelic discrimination using real-time PCR 5′ nuclease assays (Life Technologies, Grand Island, NY) on an Applied Biosystems 7300 real-time PCR (Life Technologies).

## 3. Discussion

This report summarizes the clinical course of a supratherapeutic response to warfarin in a 74-year-old white female due to impaired warfarin metabolism as a result of a CYP2C9*3*3 genotype as well as increased pharmacodynamic sensitivity due to the presence of two variant alleles of *VKORC1*. Patients who possess one or more genetic variations in *CYP2C9* and *VKORC1* are at an increased risk of adverse drug events with warfarin and require significant dose reductions to achieve therapeutic INRs [1]. Patients with the CYP2C9*3*3 and *VKORC1-1639 AA* genotypes are expected to be extremely warfarin sensitive and to require very small doses to achieve the desired therapeutic effect. Additionally, the time required to achieve a maximum, stable INR from a warfarin dosage regimen is delayed due to the impaired metabolic clearance and longer elimination half-life. It has been estimated that, with CYP2C9*3*3 genotype, patients will require at least two to four weeks to reach a maximum, stable INR from a given dose of warfarin [9]. Therefore, dosage adjustments should be made much less frequently than normal.

In this case report, the patient initially received only two mg per day for one week; yet this very conservative dose still resulted in three clinically significant hemorrhagic events and required four vitamin K antidote treatments over a period of less than two weeks after the warfarin was held. This prolonged effect was a direct result of her deficient capacity to metabolize warfarin as a consequence of possessing two *CYP2C9* variant alleles resulting in a much longer elimination half-life of warfarin. The patient also possessed two variant alleles of *VKORC1* which resulted in an increased pharmacodynamic sensitivity to the vitamin K antagonistic effects of warfarin. The combination of these two factors could have resulted in more severe outcomes. Fortunately, this patient received immediate medical care for her hemorrhagic events and appropriate vitamin K antidote treatments.

Patients with this combination of genotypes are rare, and therefore only a limited number of patients with the CYP2C9*3*3 and *VKORC1-1639 AA* genotypes receiving warfarin have been reported in the literature. The *VKORC1-1639 AA* and the CYP2C9*3*3 genotypes have been reported to occur at frequencies of 32.5% and 0.4% in a mixed ethnic population, respectively [10]. A literature search yielded four case reports (one in the form of a letter to the editor) with detailed clinical courses of five patients including demographic information, comorbidities, and concomitant medications. A summary of these case reports and the current one is presented in Table 1.

The Coumadin package insert provides ranges of expected maintenance doses based on genotypes [9]. The expected maintenance dose range in the package insert for a patient with CYP2C9*3 and *VKORC1-1639 AA* genotypes is 0.5–2.0 mg per day. This expected dose range in the package insert may be higher than the observed dose range of 0.25–1.25 mg per day in the case report patients in Table 1 because it does not incorporate additional factors (e.g., age, body size, gender, diet, ethnicity, comorbidities, and concomitant drug therapy) that are known to influence warfarin dosing. Several pharmacogenetic-based warfarin dosing nomograms which incorporate additional clinical factors are now available, and the two most commonly cited ones can be accessed at http://www.warfarindosing.org/ [10, 14]. For the present patient case, these nomograms yielded estimated therapeutic warfarin doses of 0.9 and 0.2 mg per day. Interestingly, the average of these estimated doses would be approximately 0.5 mg per day, and this was the dose the patient was stabilized on after pharmacogenetic testing.

If this patient had been genotyped for *CYP2C9* and *VKORC1* variant alleles prior to the selection of her warfarin dose, one can only speculate what actual warfarin dose would have been chosen. However, given the dosing guidelines described above, the patient would have likely received a significantly lower dose than two mg per day and may have avoided the hemorrhagic events. Due to the complications with her initial warfarin dose, she was also not optimally anticoagulated for an extended period of time which put her at increased risk of a thromboembolic event. Klein et al. reported that approximately 66% of patients of mixed ethnicities had at least one VKORC1 variant and 24% had at least one CYP2C9 variant [10]. Therefore, the majority of patients could benefit from pharmacogenetic testing prior to the initiation of warfarin therapy. Our case report details the potential challenges and risks for patients and clinicians alike in the absence of *CYP2C9* and *VKORC1* pharmacogenetic testing.

## Figures and Tables

**Table 1:** Case reports of warfarin-dosing in patients with CYP2C9*3*3 and *VKORC1-1639 AA* genotypes.

Age (yrs)	Weight (kg)	Gender	Ethnicity	Indication	Target INR	Drug Interaction(s)	Therapeutic warfarin dose (mg/d)	Reference
69	79.8	Female	Japanese	AFib	1.5–20	NR	0.5	[8]
71	82	Male	White	AFib	2.0–3.0	Amiodarone	0.9	[11]
58	70	Male	Japanese	AFib	NR	Propafenone	0.25–0.75	[12]
68	70	Female	Chinese	AFib	2.0–3.0	NR	0.63	[13]
50	88	Male	Chinese	AFib	2.0–3.0	NR	1.25	[13]
74	54	Female	White	AFib	2.0–3.0	Amiodarone/ Atorvastatin	0.5	Present case

AFib: atrial fibrillation; NR: none reported.
